# Examining DIF in the Context of CDMs When the Q-Matrix Is Misspecified

**DOI:** 10.3389/fpsyg.2018.00696

**Published:** 2018-05-11

**Authors:** Dubravka Svetina, Yanan Feng, Justin Paulsen, Montserrat Valdivia, Arturo Valdivia, Shenghai Dai

**Affiliations:** ^1^Department of Counseling and Educational Psychology, Indiana University Bloomington, Bloomington, IN, United States; ^2^Department of Statistics, College of Arts and Sciences, Indiana University Bloomington, Bloomington, IN, United States; ^3^Educational Psychology, College of Education, Washington State University, Pullman, WA, United States

**Keywords:** cognitive diagnostic models, differential item functioning, Q-matrix misspecification, test bias, validity

## Abstract

The rise in popularity and use of cognitive diagnostic models (CDMs) in educational research are partly motivated by the models’ ability to provide diagnostic information regarding students’ strengths and weaknesses in a variety of content areas. An important step to ensure appropriate interpretations from CDMs is to investigate differential item functioning (DIF). To this end, the current simulation study examined the performance of three methods to detect DIF in CDMs, with particular emphasis on the impact of Q-matrix misspecification on methods’ performance. Results illustrated that logistic regression and Mantel–Haenszel had better control of Type I error than the Wald test; however, high power rates were found using logistic regression and Wald methods, only. In addition to the tradeoff between Type I error control and acceptable power, our results suggested that Q-matrix complexity and item structures yield different results for different methods, presenting a more complex picture of the methods’ performance. Finally, implications and future directions are discussed.

## Introduction

Cognitive diagnostic models (CDMs) are a modeling approach aimed at providing examinees diagnostic information about their strengths and weaknesses relative to learning in specific domains such as mathematics. A common example in the CDM literature is its application to mathematical operations with fractions data. Here, an application of a CDM would identify for a teacher and a student a number of specific skills^1^ involved in fraction and subtraction, for example, a conversion of a whole number to a fraction, and it would provide information whether the student is deficient or has mastered this particular skill. Such information would be obtained for all hypothesized skills, assumed to underlie an assessment, and thus would provide more fine-grained information of skills and masteries, as opposed to a general estimate of the student’s (mathematics) ability (Tatsuoka, 1990, 2002; de la Torre, 2009). When applying CDMs to data, researchers (and content experts) posit a hypothesized cognitive structure, known as the Q-matrix, indicating the links between the latent skills of interest and the participant’s observable test responses. In CDMs, a Q-matrix is used to map the skill(s) necessary for correct item response onto each item.^2^ Thus, accurate Q-matrix specification is an important aspect of any CDM venture.

Despite the promise of CDMs and the significant research conducted on various models, to this point, CDMs have had relatively limited impact on actual assessments. In other words, while researchers have recognized the importance of cognitive diagnosis, the development of the models is far more advanced than test construction using the cognitive diagnosis framework (Lee et al., 2012; Hou et al., 2014). In part, this can be attributed to the relative paucity of research relating CDMs to the processes necessary for developing tests and reporting results, including examining differential item functioning (DIF). Nonetheless, creating assessments for diagnostic purposes (or any other) is not void of fairness issues. It is in this context that we situate our current research.

Differential item functioning reflects potential test bias by having different probabilities of obtaining a correct response for people with the same ability but which belong to different populations (groups). However, a deeper unfairness could be rooted in the specification of the Q-matrix, potentially further biasing score interpretations. Motivation for studying Q-matrix misspecification stems from empirical research that suggests a possibility for different groups to be subject to different Q-matrices. For example, different groups may engage in different strategies to correctly answer an item or particular skills required for typical performers may be irrelevant for those with accommodations. This kind of misspecification would result in a structurally determined form of DIF (Svetina et al., 2017). The current study approaches studying DIF within the context of CDMs, with a focus on understanding how different structures of Q-matrices impact DIF methods’ performance in finding problematic items. Specifically, our research addressed two questions:

(a) How does Q-matrix misspecification impact DIF methods’ performance to identify problematic items?
(b) How does Q-matrix misspecification in one but not both groups impact the performance of DIF methods to identify problematic items?

This paper is organized as follows. First, we provide the background for the study by discussing CDMs in general, and more specifically the model used in the current study. Additionally, we present a brief summary of the existing CDM DIF and Q-matrix misspecification literature to situate our current study. The next section describes the methodology, including the design of the simulation study and the analysis plan and outcomes. Results are then summarized in the following section, where we highlight main findings. Lastly, we discuss the implications for future research in addition to acknowledgment of the limitations.

## Background

### Cognitive Diagnostic Models

The trend in education calling for tests to provide more information at a greater detail, while requiring fewer tests (or testing times), has created a demand for test outcomes CDMs can produce. CDMs developed from the combination of various latent class modeling approaches and the cognitive psychology belief that responding correctly to individual items relies on the mastery of some set of attributes or skills (von Davier, 2008). Under this framework, a CDM requires a confirmatory loading structure – the Q-matrix – that maps the multidimensional skills required by the individual items using a complex or simple structure (Rupp and Templin, 2008).

As noted above, the Q-matrix is a loading structure that specifies which attributes are required to answer an item correctly. A typical approach to creation of the Q-matrix is to consult the experts in the content area (e.g., teachers) and cognitive psychology who would provide insights as to which skills are necessary to respond to a particular item. It has been argued that Q-matrix creation should be based at least partially based on cognitive labs, as examinees may be in a good position to hypothesize about the necessary (or used) skills in problem solving. A sample Q-matrix for four items and five attributes is presented in **Table 1**. In the Q-matrix, an entry of 1 indicates the attribute is required to correctly answer the item, while 0 indicates the attribute is not required. For example, item 1 requires only attribute 4, while item 3 requires attributes 2, 3, and 4. The probabilistic portion of the model then accounts for deviations from expected results (e.g., guessing correctly or incorrectly) by predicting the probability of mastery on the multiple attributes or dimensions designated in the loading structure.

**Table 1:** Sample Q-matrix for five attributes across four items.

		Attributes/skills (K)				
		Skill 1	Skill 2	Skill 3	Skill 4	Skill 5
Items	Item 1	0	0	0	1	0
	Item 2	1	0	1	0	0
	Item 3	0	1	1	1	0
	Item 4	0	0	1	0	1

#### DINA Model

In the current study, we employ one of the most popular and parsimonious CDMs, the deterministic inputs, noisy, “and” gate (DINA) model (Junker and Sijtsma, 2001).

Let *X_ij_* be a response of an examinee *i* to item *j*, where *X_ij_* = 1 is the correct response (0 otherwise), q-vector *q_jk_* = 1 or 0 represents the entry in the Q-matrix indicating whether attribute *k* is required by item *j*, and α*_ik_* = 1 or 0 represents if examinee *i* mastered skill *k*. Parameter η*_ij_* indicates whether examinee *i* possesses all the required attributes for item *j*, such that

(1) $$\begin{array}{c} \eta_{ij}\;=\;\prod_{K=1}^{K}\alpha_{ik}^{q_{jk}}. \end{array}$$

Parameter η*_ij_* serves in the DINA model as the “and” gate combining the deterministic outputs α*_ik_*, and the item response *X_ij_* is modeled as a noisy observation of η*_ij_*. The item response function (i.e., correct response) for the DINA model then can be expressed as:

(2) $$\begin{array}{c} P(X_{ij}=1|\alpha_{ij})\;=\;{\left(1-s_{j}\right)}^{\eta_{ij}}g_{j}^{\left(1-\eta_{ij}\right)}, \end{array}$$

where *s_j_* and *g_i_* denote slipping and guessing parameters. Parameter *s_j_* (i.e., the slipping parameter) expresses the probability of obtaining an incorrect response to item *j* when all required skills are mastered, and parameter *g_j_* (i.e., the guessing parameter) represents the probability of obtaining a correct response to item *j* when at least one required skill is not mastered.

### Differential Item Functioning (DIF)

Differential item functioning is of particular importance in the test development process because of its impact on equity in score interpretation. DIF exists when two examinees with the same ability, but who come from different groups, have different probabilities of endorsing the correct answer for an item. In DIF-type analysis, an analyst typically investigates item parameter invariance between two or more groups of examinees: a single *reference* group and one or more *focal* groups.

Furthermore, when DIF is investigated and interpreted at an item-level, the analyst may consider methods that provide information about the direction of DIF. For example, an analyst may assign *females* as a reference group, and *males* as focal. If a particular item is flagged as containing DIF, it may favor either a reference or a focal group throughout the latent continuum (known as uniform DIF). In such cases, item difficulty parameter may be shifted to the right on the scale for the *females* group when compared to the *males* group, suggesting that this item favors *male* students over *female* students along the entire proficiency scale (i.e., this item is thus more difficult for *females* than it is for *males*). This measurement variance is expressed via members of the *male* group having higher probability of success (i.e., correctly responding to an item) than members of the *female* group. Alternatively, an item may benefit a reference group at some part of the continuum, while in other parts of the latent continuum, it may favor the focal group (this is also known as nonuniform or crossed DIF).

#### DIF in CDMs

In CDMs, DIF is present when the success probability of an item is different for examinees with the same attribute mastery profile but different group membership. Specifically, in the DINA model, DIF exists when the estimated guessing or slipping parameters are different for the focal and the reference groups. However, to this point relatively few studies have considered the impact of DIF in CDMs. This is a significant weakness as it potentially exposes CDM based results to biased measurement. The few studies that have investigated DIF in the context of CDMs (Milewski and Baron, 2002; Zhang, 2006; Li, 2008; Hou et al., 2014; Li and Wang, 2015) have primarily focused their attention on comparing various methods to identifying DIF. We briefly describe this literature here in order to provide motivation for our study.

Milewski and Baron (2002) conducted a study of preliminary SAT data using DIF methodology to compare schools or states to the total population at the level of attributes of interest (as opposed to flagging poorly performing items). They used a modified rule-space model for their CDM (Tatsuoka, 1983; Tatsuoka and Tatsuoka, 1992; DiBello, 2002) and then compared several DIF methods, including *Mantel–Haenszel*, binary standardization, polytomous standardization, and ANCOVA. The authors found that the ANCOVA method was particularly sensitive to group differences, while the other methods performed fairly similarly.

Zhang (2006) focused on *Mantel–Haenszel* and SIBTEST methods when studying DIF in the DINA model. An important aspect of his study was the comparison of the conditioning variables – a typical total test score and an estimate of attribute mastery patterns, which was introduced for the first time. *Mantel–Haenszel* and SIBTEST performed similarly well in identifying uniform DIF but were unable to identify nonuniform DIF. Conditioning on attribute profiles functioned better than total score when the Q-matrix was correctly specified, for example Type I error rates in conditions with moderate DIF using profile were 0.044 and 0.043 for small and large sample size, while comparable rates when conditioning on the test scores were 0.042 and 0.055. When DIF was large, differences were more magnified such that rates of 0.051 (0.057) were found for small (large) sample size under profile and 0.071 (0.120) under test score condition variable conditions. The author, however, notes that using profile as conditioning method is more computationally demanding than using the total score, which warranted further exploration.

Similar to Milewski and Baron (2002), Li (2008) studied the impact of DIF and differential attribute functioning in the context of a modified higher order DINA model. Results suggested that model was more accurate in recovering item than attribute parameters. Further, this approach had better Type I error control and higher power than *Mantel–Haenszel*, particularly in nonuniform DIF conditions. In addition, in DIF analysis, matching on attribute profile (as opposed to total score) appeared superior in the simulation study, although in some cases using real data, inflated Type I error rates were found.

Hou et al. (2014) used a DINA model to compare the Wald test to both *Mantel–Haenszel* and SIBTEST procedures in presence of uniform and nonuniform DIF using the attribute mastery profiles as conditioning variable. Authors found that the Wald test performed better than the other methods when the proportion of DIF items was higher; however, it suffered from inflated Type I error when items poorly discriminated (Type I error rates varied, and ranged from 0.053 to 0.182 across various conditions).

Li and Wang (2015) developed a method that uniquely addressed DIF by regressing item parameters on grouping variable when more than two groups were examined. Using an LCDM and conditioning on the attribute profile, the authors found that their method performed similarly to the Wald test in terms of Type I error rates with two groups, and yielded higher power rates in both uniform and nonuniform DIF items and in situations with higher guessing and slipping parameters. When three groups were considered, the regression method produced lower Type I error rates compared to the Wald method (likely because of differences in attribute profile distributions) and similar power rates. As with other studies (e.g., Hou et al., 2014), Li and Wang (2015) found a large range of power rates, ranging from extremely (unacceptably) low rates of 0.10s to high power rates of 0.90s.

These studies have commonalities in that DIF is introduced in differing item parameters across the groups and where a single Q-matrix is considered across the groups. However, differences appear across the studies, including the choice of a conditioning variable – that is, whether to use a total score or an attribute profile as a conditioning variable, the choice of the focus. They also differed in terms of whether the focus is on differential item or skill functioning, and the choice of the methods used to detect DIF in the CDM context. However, to the best of our knowledge, no study has considered the impact of Q-matrix misspecification in CDMs while studying the impact of DIF. Given the central role of the Q-matrix in any CDM analysis, we consider this to be an important factor when examining DIF in CDMs.

### Q-matrix Misspecifications in CDMs

Traditionally, Q-matrix misspecification is defined in terms of omitting a necessary or required skill or adding an unnecessary skill in the Q-matrix (Rupp and Templin, 2008; Im and Corter, 2011; Kunina-Habenicht et al., 2012). Several scholars have studied impacts of overfitting (inclusion of a superfluous skill) and underfitting (exclusion of an essential skill) of a Q-matrix on parameter recovery and classification (e.g., Rupp and Templin, 2008; Im and Corter, 2011).

These authors found that each type of misspecification resulted in specific parameters overestimation (e.g., overfitting led to overestimation of the slipping parameter but accurate estimation of the guessing parameter). Q-matrix misspecification led to misclassification of entire classes that were related to skills omitted from particular items; however, because of the limited sizes of each class, this did not have a large overall misclassification impact. Kunina-Habenicht et al. (2012) similarly found over- and underfitting led to decent parameter recovery across complex and simple items in large samples, but poor parameter recovery for complex items in small samples. These studies suggest that misspecification can interfere with accurate parameter estimation. However, it is unclear how Q-matrix misspecification will interact with DIF, which is basis for our study design described next.

## Materials and Methods

In order to address our research questions and examine the impact of DIF on CDMs with misspecified Q-matrices, we conducted a Monte-Carlo simulation study. In what follows, we offer a rationale for the choices made in the simulation study design, including manipulated factors (and respective levels) of the simulation study, and the process of the selection/modification of item and person parameters. Lastly, we provide the analysis plan to guide our results discussion.

### Manipulated Factors

The manipulated factors fell into two categories: DIF- and CDM-related factors. We considered two DIF-related factors: DIF type (uniform DIF or nonuniform DIF) and DIF size (moderate or large). We also manipulated three CDM-related factors: position of misspecification (randomly across all items, occurs only among items measuring two or fewer attributes, or only among items measuring three or more attributes), impacted group of misspecification (misspecified in both groups, or misspecified in focal group only), and the attribute correlation (0.3, 0.5, 0.8).

### DIF-Related Factors

#### DIF Type

Similar to the IRT context, uniform and nonuniform DIF can also be differentiated in the DINA model. DIF-type is governed by the directionality of the difference in slipping and guessing parameters. Specifically, if the differences in the guessing and slipping parameters are in the same direction, uniform DIF occurs. Otherwise, nonuniform DIF occurs.

#### DIF Size

Magnitude of DIF varies across studies and its magnitude is based on the model used in the study. Generally, however, scholars examine various ranges of DIF to research the impact of DIF. In our study for DINA model, we utilized two levels of DIF (also studied in Zhang, 2006; Hou et al., 2014): moderate and large. We defined moderate DIF as difference in item parameter (Δ*s_j_* or Δ*g_j_*) equaled 0.075, while the large DIF was defined as difference of 0.100 for the focal and reference item parameters.

The combinations of two DIF-related factors resulted in 16 conditions. Following Hou et al. (2014), we illustrated the 16 DIF conditions (**Table 2**). For example, in the first row, we observe that the differences of slipping (Δ*s_j_*) and guessing parameters (Δ*g_j_*) between the focal and reference groups were in the same direction, representing uniform DIF. For each DIF size, there were two uniform DIF conditions: both Δ*s_j_* and Δ*g_j_* were positive and both Δ*s_j_* and Δ*g_j_* were negative. For each DIF size, there were six nonuniform DIF conditions: Δ*s_j_* and Δ*g_j_* had opposite signs or one of the deltas was zero. Therefore, there were eight DIF conditions for each DIF size, resulting in 16 DIF conditions in total. In addition, we simulated a baseline (no DIF) condition where the guessing and slipping parameters for the focal and reference groups were the same.

**Table 2:** Summary of DIF conditions^∗^.

DIF type	DIF size	Δ*g_j_* (*g*_F_*_j_* – *g*_R_*_j_*)	Δs*_j_* (*s*_F_*_j_* – *s*_R_*_j_*)
Uniform DIF	Moderate	+0.075	+0.075
		–0.075	–0.075
	Large	+0.10	+0.10
		–0.10	–0.10
Nonuniform DIF	Moderate	+0.075	–0.075
		+0.075	0
		–0.075	+0.075
		–0.075	0
	0	–0.075
	0	+0.075
	Large	+0.10	–0.10
		+0.10	0
		–0.10	+0.10
		–0.10	0
	0	–0.10
	0	+0.10

*^∗^DIF combinations were modeled after Hou et al. (2014).*

### CDM-Related Factors

Given the focus of our study was to understand the performance of the methods in detecting DIF in CDMs, we manipulated several factors related to CDM construction. That is, we examined the impact of Q-matrix structure and its misspecification, as well as the attribute correlation on methods’ ability to identify DIF.

#### Position of Misspecification

The Q-matrix misspecification occurred at one of the three levels: at random across all items, only among items measuring one or two attributes, or only among items measuring three or more attributes. Our rationale for including this factor was based on the idea that items which require more attributes are more likely to be misspecified, as that formation of Q-matrix is a complex process which may leave out some attributes while potentially including unnecessary ones. As scholars have previously suggested, Q-matrix misspecification (overfitting or underfitting) may occur for a number of reasons, including the fact that skills may be hierarchically related (yet not accounted for in the Q-matrix construction), and a failure to account for alternative strategies when solving items (e.g., Rupp and Templin, 2008; Im and Corter, 2011).

#### Impacted Group of Misspecification

The Q-matrix that was misspecified impacted either both reference and focal groups (in the same way), or only the focal group. We utilized those two levels because in some situations, it is possible that the focal and reference group may have a different Q-matrix for the same test, yet in empirical research, both groups would have a single Q-matrix.

#### Attribute Correlation

The correlations among attributes in CDMs literature ranged from small to large (e.g., Zhang, 2006; Cui et al., 2012), even though studies typically use a fixed level of attribute correlation 0.5 (e.g., Henson and Douglas, 2005; Chiu, 2013; Bradshaw and Templin, 2014). However, applications of hierarchical CDMs suggest that attributes may be related differently. Thus, in order to examine the impact of the correlation among attributes, we used three levels of attribute correlation in our study: 0.3, 0.5, and 0.8.

In addition to the six conditions combined from the two Q-matrix misspecification factors, a baseline condition without Q-matrix misspecification was also included in our study. The Q-misspecification factors and the attribute correlation were fully crossed, thus creating a total of 21 CDM-related conditions.

### Data Generation

In the simulation, we assumed that 2000 simulees (*N* = 1000 in reference and focal groups, respectively) responded to 31 dichotomously scored items measuring between one and five attributes. Data were generated using the DINA model (see Equation 2) with randomly chosen item parameters that align with empirically found values (de la Torre and Douglas, 2004) and the combination of factors previously discussed. Specifically, guessing and slipping parameters for the baseline conditions were randomly drawn from ∼*U*[0.1, 0.3]. The mean slipping parameter was 0.226 (ranging from 0.103 to 0.291), and the mean for the guessing parameter was 0.182 (ranging from 0.102 to 0.298). These values are comparable to those found in the literature using DINA model (e.g., Zhang, 2006; de la Torre, 2009; Li and Wang, 2015).

In order to simulate DIF, we selected six items^3^ (about 20%) at random and increased (decreased) the baseline values of slipping/guessing parameters per simulation design. Q-matrix misspecification was modeled such that 10% of the Q-matrix entries were randomly misspecified by changing the original q-entries from 0 to 1 or from 1 to 0.

Data were simulated in R, Version 3.3.2 (R Core Team, 2016). The R package difR was used to do DIF analysis using MH and Logistic method (Magis et al., 2010), and the Wald test was conducted using the GDINA package in R (Ma and de la Torre, 2017). All study code is available upon request. Based on the study design – the DIF-related factors fully crossed with the CDM-related factors, resulted in 357 conditions.

### Analysis and Outcomes

Each condition was replicated 100 times and results were averaged across the 100 replications. We compare the performance of the three DIF detection methods in terms of Type I error and power. Type I error in this context indicates that an item is flagged as having DIF when it does not. Power rates are defined by the number of times an item is correctly flagged as having DIF. For each of the 25 items that do not have DIF, the Type I error rate is defined as the percentage of times the item is detected as DIF out of the 100 replications. In terms of power rates, for each of the six items that have DIF, the power rate is defined as the percentage of times the item is correctly detected as DIF out of the 100 replications. These are reported as percentages in the table below averaged across replication.

## Results

We present the results in two sections, beginning with the graphical representations of the overall results for large DIF (i.e., Δ*g_j_* and Δ*s_j_* = 0.10) conditions across the three studied methods.^4^ These overall rates represent an average across all items within a replication, which are in turn averaged across 100 replications within a condition. The second section discusses results by focusing on methods’ performance across items with different characteristics. Specifically, we report results for items that had relatively small number of attributes associated with them (items with one or two attributes) and for items with larger number of attributes (three or more). Within each section, results are shown for Type I error, first, followed by power rates.

### Overall Results

**FIGURE 1:**
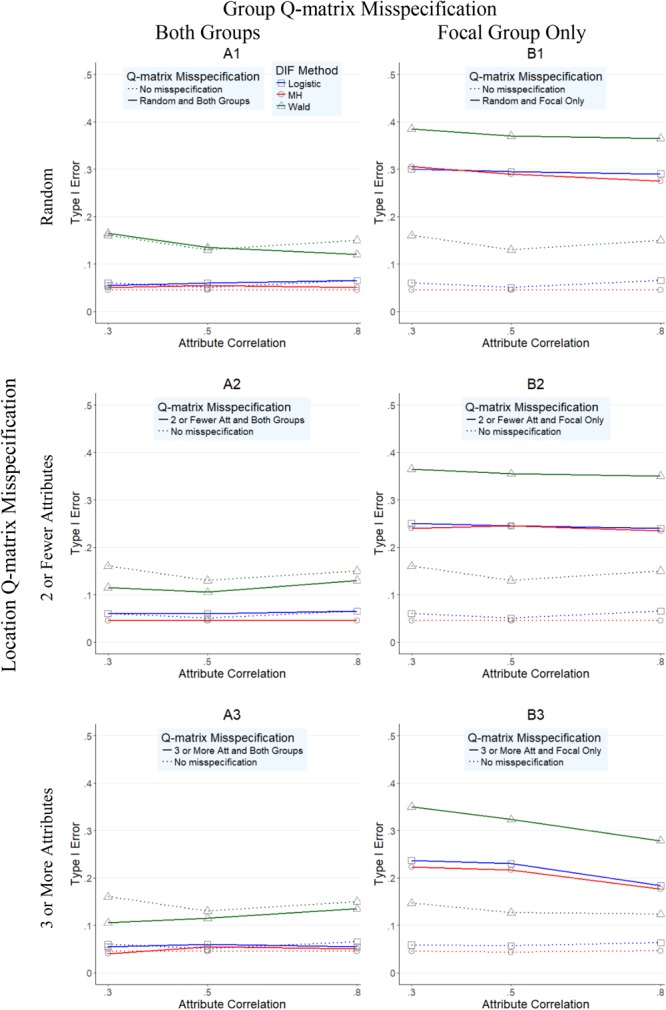
Type I error rates for DIF methods across attribute correlations when large uniform DIF is present.

**FIGURE 2:**
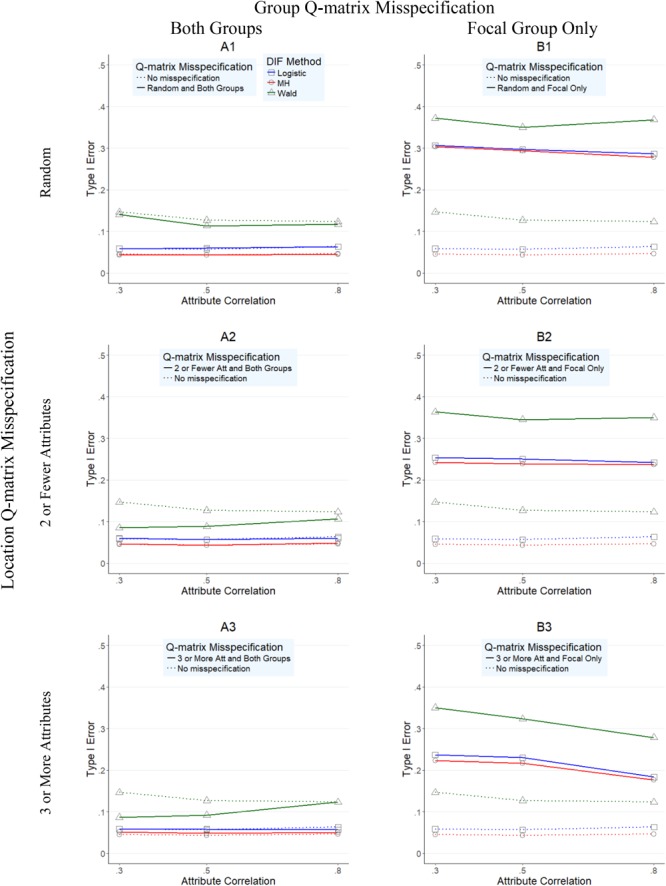
Type I error rates for DIF methods across attribute correlations when large nonuniform DIF is present.

**FIGURE 3:**
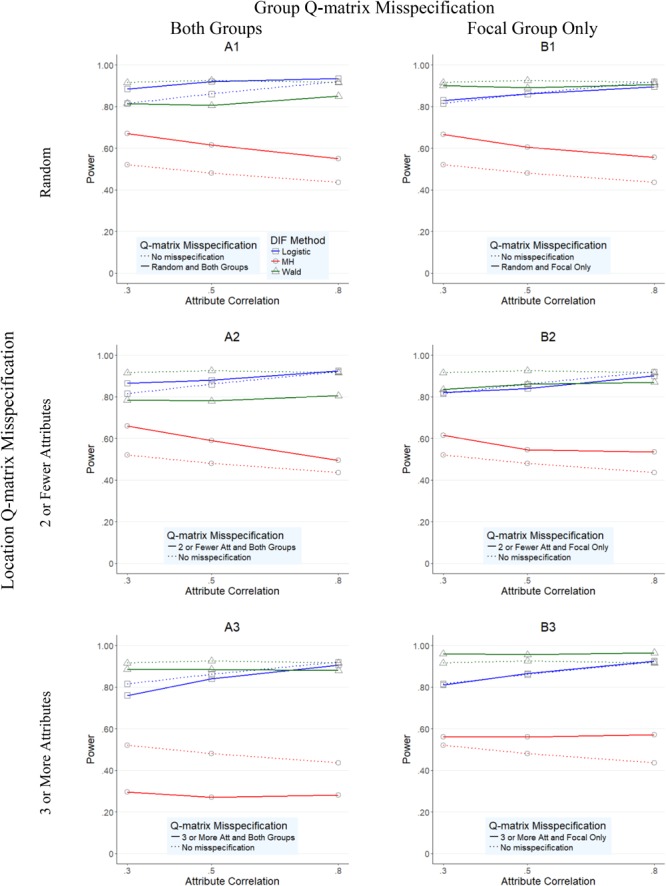
Power rates for DIF methods across attribute correlations when large uniform DIF is present.

**FIGURE 4:**
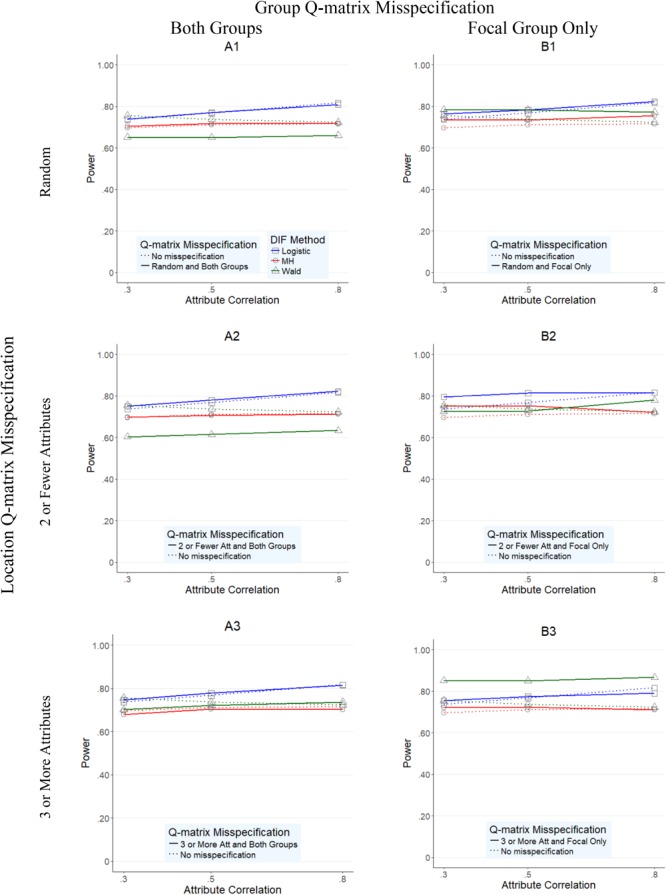
Power rates for DIF methods across attribute correlations when large nonuniform DIF is present.

**Figures 1–4** present the average overall Type I error and power rates for uniform and nonuniform large DIF. In all figures, columns represent conditions in which the Q-matrix was misspecified in (a) both reference and focal groups (left column) and (b) only in focal group (right column). Rows of the figures represent the location of the Q-matrix misspecification, such that in the top row, misspecification is random (i.e., random Q-matrix entries are misspecified); in the middle row, misspecification is concentrated in items that required only two or one skills; and in the bottom row, the misspecification of the Q-matrix is simulated in items which require three or more attributes. Within each figure, dashed lines indicate the baseline rates (i.e., no Q-matrix misspecification), while solid lines represent obtained results for various combinations of manipulated factors. In addition, each of the three methods is represented by color and mark, such that logistic regression is indicated by blue squares, *Mantel–Haenszel* by red circles, and Wald by green triangles.

**Figure 1** illustrates the Type 1 error rates for conditions with uniform large DIF across different attribute correlations (*x*-axis) for all three methods. As noted in the left column of **Figure 1**, when both reference and focal groups were affected by Q-matrix misspecification (A1–A3 graphs), *Mantel–Haenszel* and logistic regression methods outperformed the Wald method. Specifically, while *Mantel–Haenszel* had the lowest Type I error rates (average of 0.048, and with a range from 0.04 to 0.06), closely followed by logistic regression rates (average of 0.059, and with a range between 0.05 and 0.07), the Wald method yielded inflated Type I error rates all above 0.10 (average of 0.125, and with a range from 0.07 to 0.39). When the Q-matrix was misspecified only for the focal group, all three methods yielded inflated Type I error rates even though the *Mantel–Haenszel* and logistic regression yielded lower rates than the Wald method. Across all three methods, the most inflated rates were found in conditions when random misspecification was simulated (B1 graph), where rates reached as high as 0.39, 0.31, and 0.30 for Wald, *Mantel–Haenszel*, and logistic regression, respectively.

**Figure 2** presents the Type I error rates for conditions with nonuniform large DIF across different attribute correlations (*x*-axis) for all three methods. Very similar findings (and patterns) to those in **Figure 1** were observed in **Figure 2**, suggesting again that the Wald method performed worse than the other two methods. However, two additional observations were made. First, across all conditions, Type I error rates were higher for all methods under nonuniform (**Figure 2**) than under uniform (**Figure 1**) DIF. Second, the relative performance of the methods was more similar under nonuniform DIF than under uniform DIF, as indicated by closer proximity of the red, blue, and green lines.

Overall average power rates for the three methods are reported in **Figures 3, 4**. Unlike Type I error results, methods performed rather differently for conditions under uniform and nonuniform DIF. As **Figure 3** shows, *Mantel–Haenszel* reported the lowest power rates across the studied conditions when uniform large DIF was present; its power rates never exceeded 0.72 (average of 0.535, and with a range between 0.25 and 0.72) across any condition. Wald and logistic regression methods, however, yielded reasonable power rates, typically above 0.80.

Specifically, when both groups were impacted by the misspecified Q-matrix (A1–A3 graphs in **Figure 3**), logistic regression yielded the highest power rates, with an average of 0.879 (and range from 0.72 to 0.97) across conditions, and in particular high power rates for conditions when misspecification was either random (A1 graph in **Figure 3**; average power of 0.913, range of 0.85–0.94) or in items with two or one attribute (A2 graph in **Figure 3**; average power of 0.890, range of 0.85–0.95) across the attribute correlation levels. In the same conditions, the Wald method yielded slightly lower, but still relatively acceptable power rates which averaged of 0.823 and ranged from 0.71 to 0.88.

Furthermore, when Q-matrix misspecification was located in items with three or more attributes (A3), logistic regression and the Wald method performed similarly, although the Wald method yielded higher rates than logistic regression when attribute correlations were at 0.30 or 0.50, while Wald method yielded higher power rates at 0.80 correlation. Specifically, the Wald method yielded power rates of 0.885, 0.885, and 0.880 across three levels of attribute correlation (0.30, 0.50, and 0.80), while logistic regression yielded 0.760, 0.840, and 0.905, respectively. *Mantel–Haenszel* never exceeded 0.310 in the comparable conditions.

When the Q-matrix was misspecified for the focal group only (B1–B3 graphs in **Figure 3**), similar patterns of results to those of A1–A3 were found. Namely, *Mantel–Haenszel* yielded lower power rates than logistic regression or the Wald method with an average of 0.579 and range from 0.50 to 0.68 across any condition, while logistic regression and the Wald method yielded rates with averages (ranges) of 0.860 (0.76–0.94) and 0.904 (0.81–0.97), respectively. In addition, compared to A1–A3, Wald method and logistic regression yielded power rates more similar to each other, suggesting fewer differences in performance between these two methods when Q-matrix misspecification occurred only in the focal group.

When nonuniform DIF was present (**Figure 4**), logistic regression and Wald method generally yielded lower power rates when compared to the uniform condition (**Figure 3**) counterparts, although their rates were still reasonably high with overall averages of 0.785 and 0.728, respectively. In most conditions (except B3 graph), logistic regression yielded equally high or the highest power rates when compared to Wald method and *Mantel–Haenszel*, as indicated by the position of the blue line relative to red or green. Specifically, when both groups were impacted by the misspecified Q-matrix (A1–A3 graphs in **Figure 4**), logistic regression yielded the highest power rates, with an average of 0.879 (and range between 0.72 and 0.97) across conditions, compared to the average (range) power rate yielded by Wald method and MH, 0.664 (0.20–0.90) and 0.704 (0.23–1.00), respectively.^5^ When only the focal group was impacted, the three methods tended to perform reasonably similar to each other under conditions where the Q-matrix was misspecified at random or in items with two or fewer attributes (B1 and B2 graphs), as indicated by the red, blue, and green lines being relatively close to each other. The largest difference in performance was noted in conditions where Q-matrix misspecification occurred in items with three or more attributes, where the Wald method, which yielded generally high rates across conditions, outperformed the other two methods. In those conditions, Wald was the only method with the average power rates of above 0.80 across all three attribute correlation levels (851, 0.850, and 0.867 for 0.30, 0.50, and 0.80 correlation levels, respectively).

### Results for Items With Fewer and Larger Number of Attributes

Overall rates, as presented in the aforementioned figures, provide information about general trends across the simulated conditions. We further report results by examining the impact of Q-matrix structure – namely, by examining the performance of the methods in identifying DIF for different types of items. In the following tables, we report Type I error and power rates for items with only one or two attributes (indicated as 2 or Fewer K), and those with three or more attributes (3 or More K). This allows us to examine at a more detailed level how particular characteristic of an item (i.e., how many attributes are assumed to be required for an item) affects the performance of the methods to flag problematic items.^6^

**Table 3** presents results of the average Type I error rates for uniform [panel (a)] and nonuniform [panel (b)] DIF conditions where attribute correlation was 0.50 and simulated DIF was large. As previously suggested, Type I error results for moderate DIF and other examined levels of attribute correlations yielded similar patterns and thus were not reported here (complete results for Type I error and power rates per item are available per request).

**Table 3:** Average Type I error rates for conditions with attribute correlation of 0.50 and large DIF.

Group misspecification	Position misspecification	Mantel–Haenszel		Logistic		Wald	
		2 or Fewer K	3 or More K	2 or Fewer K	3 or More K	2 or Fewer K	3 or More K
**Panel (b): Uniform DIF**		
Neither		0.05	0.04	0.06	0.04	0.14	0.12
Both groups	At random	0.05	0.05	0.08	0.05	0.16	0.11
	2 or Fewer	0.05	0.04	0.07	0.05	0.12	0.09
	3 or More	0.06	0.05	0.07	0.05	0.12	0.10
Focal only	At random	0.40	0.19	0.40	0.19	0.47	0.28
	2 or Fewer	0.46	0.05	0.45	0.06	0.59	0.14
	3 or More	0.06	0.37	0.07	0.37	0.17	0.48
**Panel (a): Nonuniform DIF**		
Neither		0.05	0.04	0.07	0.05	0.14	0.11
Both groups	At random	0.05	0.04	0.07	0.05	0.12	0.11
	2 or Fewer	0.05	0.04	0.06	0.05	0.09	0.09
	3 or More	0.05	0.05	0.06	0.05	0.10	0.09
Focal only	At random	0.40	0.19	0.41	0.19	0.45	0.26
	2 or Fewer	0.44	0.05	0.46	0.06	0.57	0.14
	3 or More	0.05	0.37	0.08	0.37	0.16	0.47

*Under *2 or Fewer*: *K =* average Type I error rates for items with two or fewer (i.e., one) attributes. Under *3 or More*: *K* = average Type I error rates for items with three or more attributes. Under *Group Misspecification*: Neither = no group misspecification present, Both Groups = misspecification in the Reference and Focal Groups, Focal only = misspecification in Focal group only. Under *Position Misspecification*: at random = misspecification at random across all 31 items. Two or fewer = misspecification of Q-matrix entries for items 1–15 (at random) as those items were associated with two or one attribute. Three or more = misspecification of Q-matrix entries for items 16–31 (at random) as those items were associated with at least three attributes.*

As observed in **Table 3**, panel (a), *Mantel–Haenszel* and logistic regression yielded Type I error rates around 0.05 when Q-matrix was not misspecified (0.04 for items of 2 or Fewer K and 0.06 for items with 3 or More K). When Q-matrix was misspecified in both reference and focal groups, Type I error rates were lower when items had larger number of attributes associated with them (under 3 or More K), although in *Mantel–Haenszel*, the difference was marginal. In these conditions, regardless of the location of misspecification (e.g., random or for items with fewer/more attributes under *Position Misspecification* column), *Mantel–Haenszel* and logistic regression yielded acceptable Type I error rates, ranging from 0.04 to 0.08. The Wald method yielded inflated Type I error rates of about 0.12 average with a range of 0.09 to 0.16 for either items with 3 or More K or items with 2 or Fewer K.

When misspecification was located in the focal group only, all three methods reported unacceptably inflated Type I error rates with only a few exceptions. Namely, when Q-matrix misspecification was only in the focal group and misspecification was concentrated in items with two or fewer attributes, both *Mantel–Haenszel* and logistic regression reported reasonable Type I error rates for items with larger number of attributes (0.05 and 0.06, respectively, under respective 3 or More K). Similarly, when Q-matrix misspecification was concentrated in items with larger number of attributes, appropriate Type I error rates for items with fewer number of attributes were observed (0.06 and 0.07, respectively under 2 or Fewer K). In all other conditions, for all three methods, Type I error rates were unacceptably high, typically in the 0.40s.

As noted in **Table 3**, panel (b), similar magnitudes of Type I error rates as well as patterns for nonuniform DIF were observed. Here again, we observed *Mantel–Haenszel* and logistic regression yielded more acceptable Type I error rates for conditions in which the Q-matrix was correctly specified or misspecified for both groups. The Wald method again yielded inflated Type I error rates, with an average of over 0.22 and range of 0.09–0.57 regardless of how many attributes items were associated with. *Mantel–Haenszel* and logistic regression yielded somewhat lower Type I error rates, but generally followed in similar pattern as the Wald method – the worst *Mantel–Haenszel* and logistic regression performances were again found in conditions where only the focal group was impacted by Q-matrix misspecification, in particular for items with fewer attributes (2 or Fewer K).

**Table 4** presents results of the average power rates for uniform [panel (a)] and nonuniform [panel (b)] DIF conditions where attribute correlation was 0.50 and simulated DIF was large. As previously suggested, results for moderate DIF and other examined levels of attribute correlations yielded similar results and thus were not reported here. As noted in panel (a) of **Table 4**, the three methods performed differently across various levels of Q-matrix specification when DIF was uniform. Specifically, when no Q-matrix misspecification was modeled, logistic regression and Wald reported reasonably high power rates (in range of 0.80–0.96), compared to Mantel–Haenszel (0.44 and 0.52 for items with 2 or Fewer K and 3 or More K, respectively).

**Table 4:** Average power rates for conditions with attribute correlation of 0.50 and large DIF.

Group misspecification	Position misspecification	Mantel–Haenszel		Logistic		Wald	
		2 or Fewer K	3 or More K	2 or Fewer K	3 or More K	2 or Fewer K	3 or More K
**Panel (a): Uniform DIF**		
Neither		0.44	0.52	0.80	0.93	0.96	0.89
Both groups	At random	0.71	0.52	0.90	0.93	0.87	0.74
	2 or Fewer	0.65	0.53	0.84	0.92	0.86	0.70
	3 or More	0.45	0.10	0.81	0.87	0.96	0.81
Focal only	At random	0.74	0.47	0.86	0.86	0.99	0.80
	2 or Fewer	0.66	0.43	0.81	0.87	0.96	0.75
	3 or More	0.42	0.69	0.78	0.95	0.95	0.96
**Panel (b): Nonuniform DIF**		
Neither		0.71	0.71	0.75	0.78	0.79	0.69
Both groups	At random	0.70	0.73	0.75	0.79	0.70	0.60
	2 or Fewer	0.68	0.73	0.74	0.82	0.70	0.53
	3 or More	0.69	0.72	0.73	0.82	0.80	0.64
Focal only	At random	0.78	0.69	0.82	0.74	0.89	0.68
	2 or Fewer	0.79	0.71	0.82	0.81	0.84	0.62
	3 or More	0.70	0.74	0.75	0.80	0.80	0.91

*Under *2 or Fewer*: *K =* average Type I error rates for items with two or fewer (i.e., one) attributes. Under *3 or More*: *K* = average Type I error rates for items with three or more attributes. Under *Group Misspecification*: Neither = no group misspecification present, Both groups = misspecification in the Reference and Focal Groups, Focal only = misspecification in Focal group only. Under *Position Misspecification*: At random = misspecification at random across all 31 items. Two or fewer = misspecification of Q-matrix entries for items 1–15 (at random) as those items were associated with two or one attribute. Three or more = misspecification of Q-matrix entries for items 16–31 (at random) as those items were associated with at least three attributes.*

When reference and focal groups contained Q-matrix misspecification, logistic regression reported the highest power rates (0.81–0.93), followed by the Wald method (0.70–0.96) and *Mantel–Haenszel*, which reported generally unacceptably low power rates (0.10–0.71). In conditions where the Q-matrix was misspecified in the focal group only, the Wald method and logistic regression similarly outperformed *Mantel–Haenszel*. An interesting observation was noted about logistic regression and Wald method performance. Namely, in most cases, unlike logistic regression (which yielded increased or equal power rates), increase in the number of attributes (i.e., from 2 or Fewer K to 3 or More K) resulted in poorer (lower) power rates in Wald (and *Mantel–Haenszel*). This decrease was noted across most conditions regardless of the location or group Q-matrix misspecification – one exception was in condition where the Q-matrix was misspecified only in the focal group and in conditions where misspecification occurred only in items with 3 or More K. In those cases, power rates increased from 0.42 to 0.69 for *Mantel–Haenszel*, and 0.95 to 0.96 for the Wald method.

As noted in panel (b) of **Table 4**, nonuniform DIF conditions resulted in lower power rates for logistic regression and Wald method (from about 0.80 and 0.90s down to about 0.70s); *Mantel–Haenszel*, however, yielded results more comparable to the other two methods than in uniform conditions. Power rates across Q-matrix misspecification and location were generally lower in presence of nonuniform DIF, typically ranging in the 0.70s. Further, when both reference and focal groups had misspecified Q-matrix, logistic regression (and *Mantel–Haenszel*) yielded higher power rates for items with larger number of attributes, while the Wald method yielded higher power rates for items with two or fewer attributes, on average.

However, when Q-matrix misspecification was present in the focal group only, all three methods tended to be more successful in flagging problematic items (i.e., higher power rates), for items with fewer number of attributes than when Q-matrix misspecification was either random or concentrated in items with two or fewer number of attributes. Higher power rates were observed in all three methods when misspecification was concentrated in items with three or more attributes. Specifically, in these conditions, Wald method yielded the highest power rates of 0.80 and 0.91 (for fewer and more attributes, respectively), while comparable rates for logistic regression and *Mantel–Haenszel* were 0.75 and 0.80, and 0.70 and 0.74, respectively.

## Discussion

Cognitive diagnostic models are considered a promising methodology that allows more access to knowledge about student performance without necessarily increasing the test time (Hou et al., 2014). This area of research is currently gaining a lot of attention in part due to the educational climate which makes use of models to provide insight into students’ strengths and weaknesses. As with any assessment context that produces some type of scores used in high-stakes decision making, valid inferences are important. Thus, our study contributes to better understanding of how methods to detect DIF perform in the context of CDMs.

An important contribution of the study is the understanding how methods perform in detecting DIF under different Q-matrix structures including when the Q-matrix was misspecified at various degrees. Broadly, results in the current study suggested that while *Mantel–Haenszel* and logistic regression typically yielded reasonable Type I error rates, the Wald method yielded inflated Type I error rates across most conditions. Further, all methods’ performance deteriorated (i.e., higher Type I error rates) when only one group modeled misspecified Q-matrix. *Mantel–Haenszel* tended to yield the lowest power rates among the three methods, while the Wald method performed similarly to logistic regression and yielded reasonably high power rates across conditions. The implication here is that different methods performed more favorably under different conditions and that a researcher should take into account the potential tradeoff between acceptable Type I error and power rates.^7^

As with any simulation study, the settings we considered were necessarily limited, and other contexts may produce different results. For example, in our Q-matrix construction, we only considered items with up to five attributes. While this is not unusual when studying parsimonious CDMs, empirical applications of models such as reparameterized RUM or Rule Space methodology often employ a dozen or more attributes (e.g., Tatsuoka et al., 2004; Svetina et al., 2017). For example, in a study aimed at investigating and comparing eighth-graders’ mathematics performance across countries, Tatsuoka et al. (2004), together with content experts, developed a Q-matrix that contained 27 mathematics attributes that spanned across three main skill groups (content, process, and skill type).

Additionally, Q-matric complexity exponentially increases as the number of attributes increase, and thus the performance of DIF methods studied here (i.e., MH, logistic regression, or Wald) is unknown in such contexts. In addition, while our design allowed for differing correlation levels among the attributes (at low, moderate, and high levels), our design assumed that a select level of correlation was constant between all pairs of attributes. This may not work in all applications, however, as some attributes may be more strongly related than others. In a similar vein, researchers may hypothesize that some skills are related hierarchically to other skills and thus, the Q-matrix structure may result in different correlations among the attributes. Examples of several constructs, including reading comprehension and mathematics, can be found where CDMs fit to the analyses allowing for hierarchical attribute models (e.g., Gierl et al., 2008, 2010; Wang and Gierl, 2011).

In addition to addressing the above-mentioned limitations, future work could also consider contexts in which more groups or smaller sample sizes were included, as the current study considered only the large sample size of 1000 per group and just two groups. More so, in the current study, performance of the methods was evaluated via Type I errors (i.e., an item is incorrectly flagged as having DIF when the item was simulated as invariant) and power (i.e., an item is correctly flagged as having DIF as item was simulated to be noninvariant). It would be useful to explore further impact of manipulated factors on the precision or accuracy of mastery classification. In other words, CDMs provide item parameter estimates, which can help understand the precision of model estimation, but they also provide information regarding the mastery of examinees who in turn can be classified which is generally a benefit of using a CDM over a traditional statistical model. In other words, the utility of fitting CDMs is to potentially better understand the strengths and weaknesses of the examinees. One aspect in the CDM literature that deals with this is the idea of examinee classification, and factors that impact it. Our study is currently being extended to examine the factors that impact the precision of classification of examinees. Despite the limitations, the current work provides further insight into the performance of popular DIF methods in a context that is still relatively unexplored, yet which may potentially make big impacts in educational research given the current accountability era.

## Author Contributions

DS developed the original idea, conducted the data analyses and interpretation, performed majority of the writing, guided the overall research process, and contributed to editing. YF contributed to conceptualizing idea and data analyses, wrote some portions of the manuscript, and contributed to editing. JP contributed to conceptualizing idea, wrote parts of manuscript (in particular in background section), and contributed to editing. MV contributed to data interpretation and formatting, and contributed to editing. AV contributed to data analysis, in particular with data generation, and contributed to editing. SD contributed to conceptualizing the original idea, graphing, and editing.

## Conflict of Interest Statement

The authors declare that the research was conducted in the absence of any commercial or financial relationships that could be construed as a potential conflict of interest.

## Footnotes

1 Skills, attributes, or processes are used interchangeably in the literature and are used as such in the current manuscript. These skills or attributes represent the cognitive processes hypothesized in the Q-matrix which items require for successful outcome (i.e., correct answer), in other words, they indicate examinee’s knowledge structures.

2 Some CDMs, including the reparameterized unified model (RUM), include a parameter that allows for unspecified, supplemental ability to account for participants’ correct response. Even so, an item with a high level of this parameter would be flagged for further revision as it is not functioning in a desired manner.

3 These six items were then fixed across conditions to have DIF, although DIF magnitude and directionally was changed according to the study design.

4 Similar results were found for comparable conditions in presence of moderate DIF and are thus not reported here. Complete results can be obtained by request.

5 The low power rates (as indicated by low rate in the range) occurred in only a handful of conditions, mostly related to DIF being modeled in differential guessing parameter (only), and when Q-matrix misspecification was simulated for both groups and at random or for items with two or fewer attributes. Given that figures represent the overall average, and since remaining conditions had relatively high power rates, the presence of a handful of low power values did not affect the overall rates as much.

6 Items were grouped together based on the original Q-matrix. This meant that in the baseline Q-matrix (prior to any misspecification) items 1–15 were simulated to have only 1 or 2 attributes associated with them (labeled as two or fewer) and were thus grouped together for purposes of reporting in tables, and items 16–31 were grouped together to represent items with three or more attributes. The rates reported are averages across these item groups.

7 We note that outside simulation, knowledge of the models’ performance in terms of control of Type I errors or power is unknown; however, we agree with Madison and Bradshaw (2013) who suggested that researchers interested in designing a test that would ultimately employ a CDM should perform some type of power analysis (in this context, the accuracy and reliability of classification). The authors developed a tool called Q^∗^Power in attempt to address this issue, which can be freely accessed at http://www.lainebradshaw.com/qpower/.
